# Genotyping-by-sequencing (GBS), an ultimate marker-assisted selection (MAS) tool to accelerate plant breeding

**DOI:** 10.3389/fpls.2014.00484

**Published:** 2014-09-30

**Authors:** Jiangfeng He, Xiaoqing Zhao, André Laroche, Zhen-Xiang Lu, HongKui Liu, Ziqin Li

**Affiliations:** ^1^Inner Mongolia Academy of Agriculture and Husbandry ScienceHohhot, China; ^2^Lethbridge Research Centre, Agriculture and Agri-Food CanadaLethbridge, AB, Canada

**Keywords:** genomic selection (GS), genotyping-by-sequencing (GBS), marker-assisted selection (MAS), next-generation sequencing (NGS), single nucleotide polymorphism (SNP)

## Abstract

Marker-assisted selection (MAS) refers to the use of molecular markers to assist phenotypic selections in crop improvement. Several types of molecular markers, such as single nucleotide polymorphism (SNP), have been identified and effectively used in plant breeding. The application of next-generation sequencing (NGS) technologies has led to remarkable advances in whole genome sequencing, which provides ultra-throughput sequences to revolutionize plant genotyping and breeding. To further broaden NGS usages to large crop genomes such as maize and wheat, genotyping-by-sequencing (GBS) has been developed and applied in sequencing multiplexed samples that combine molecular marker discovery and genotyping. GBS is a novel application of NGS protocols for discovering and genotyping SNPs in crop genomes and populations. The GBS approach includes the digestion of genomic DNA with restriction enzymes followed by the ligation of barcode adapter, PCR amplification and sequencing of the amplified DNA pool on a single lane of flow cells. Bioinformatic pipelines are needed to analyze and interpret GBS datasets. As an ultimate MAS tool and a cost-effective technique, GBS has been successfully used in implementing genome-wide association study (GWAS), genomic diversity study, genetic linkage analysis, molecular marker discovery and genomic selection under a large scale of plant breeding programs.

## INTRODUCTION

Plant breeding can be accomplished through two major strategies, classical breeding and molecular breeding. Classical plant breeding uses the deliberate interbreeding of closely related individuals to produce new cultivars with desirable traits. As it needs a long period and several generations to select and evaluate useful genotypes, classical breeding could be limited to address global food security and meet the increasing requirements of food demands (Tester and Langridge, 2010). Molecular plant breeding is the applications of molecular biology or biotechnology to improve or develop new cultivars, which includes two major approaches, marker-assisted selection (MAS) and genetic transformation (Moose and Mumm, 2008). At moment, the application of genetic transformation (or genetic engineering) is seriously hindered because there is controversy on food safety and environmental impacts over any genetically modified (GM) crop (Nicolia et al., 2014). MAS is a process whereby molecular markers are used for the indirect selection on traits of interest in crops. As a critical and effective method, MAS has been widely applied in plant breeding to enhance crop yield, quality, and tolerance to biotic or abiotic stresses. Recent advance of genotyping-by-sequencing (GBS) offers an ultimate MAS tool to accelerate plant breeding and crop improvement.

## MOLECULAR MARKERS

Plant molecular breeding has advanced so rapidly that several types of molecular markers have been developed and used for decades. The restriction fragment length polymorphism (RFLP) was firstly applied as DNA markers in plant genotyping (Botstein et al., 1980). RFLP technique is useful in the construction of genetic linkage maps, but it is challenged by the complicated hybridization, radioactivity, and time consuming and limited by the number of available probes (Bernatsky and Tanksley, 1986). With further advance of biotechnology, several types of PCR-based markers were developed and used in plant breeding programs. These PCR-based markers mainly include random amplification of polymorphic DNA (RAPD; Williams et al., 1990), sequence characterized amplified region (SCAR; Paran and Michelmore, 1993), cleaved amplified polymorphic sequences (CAPS; Konieczny and Ausubel, 1993), simple sequence repeats (SSRs; Litt and Luty, 1986; Salimath et al., 1995), amplified fragment length polymorphisms (AFLPs; Vos et al., 1995), and direct amplification of length polymorphisms (DALP; Desmarais et al., 1998). Compared to RFLP, all these PCR-based markers are amplified form individual genomic sequences under a small scale, relatively inexpensive and less time-consuming.

In combination with the genome and expressed sequence tags (ESTs) in model plant species (Adams et al., 1991), Sanger sequencing throughput was improved to accelerate the identification of variations at the single base pair resolution (Wang et al., 1998). The use of single nucleotide polymorphisms (SNPs; Lander, 1996) as DNA markers for plant genotyping has increased the potential to score variation in specific DNA targets. More importantly, the information on potentially millions of genome-wide SNPs or small insertion-deletions and their surrounding sequences sets the foundation of high-throughput genotyping. Over the past 10 years, SNP-based marker techniques have been improved in marker density and, if compared with the earlier genotyping approaches, the costs and time on SNP discoveries have been significantly reduced. Among them, the fluorescent detection of SNP-specific hybridization probes on PCR products, including Taqman, Molecular Beacons, and Invader, is the most commonly used system (Tapp et al., 2000; Prince et al., 2001; Livak, 2003; Storm et al., 2003; Olivier, 2005; Ragoussis, 2006). In addition, the homogeneous mass-extend (hME) assay also uses SNP-specific PCR primer extension products but results are read on a MALDI-TOF mass spectrophotometer (Ragoussis, 2006). All these techniques can acquire 100–1000s of SNPs on a daily basis. With the increasing requirement of higher throughput data, the Taqman and Invader technologies have been significantly improved by enhancing the microtiter plates from 96 to 1536 wells (Procunier et al., 2009).

Molecular markers are extremely useful in plant genetics and breeding. Markers are prerequisite for gene mapping and tagging, segregation analysis, genetic diagnosis, forensic examination, phylogenetic analysis and numerous biological applications (Semagn et al., 2006; Lam et al., 2010; Singh et al., 2010; Sonah et al., 2011a). Although several types of molecular markers have been developed and are routinely being used in plant breeding, most of these marker systems are restricted in their applications because of the limitation on their availability and the high cost of analyses conducted on a large scale. Among various types of molecular markers (Agarwal et al., 2008; Sonah et al., 2011b), SNPs are the most abundant in a genome and suitable for analysis on a wide range of genomic scales (Rafalski, 2002; Zhu et al., 2003). However, the development of high-throughput genotyping platforms for large numbers (thousands to millions) of SNPs has proved to be relatively time-consuming and costly. Typically, a fairly large sequencing effort is devoted to identify polymorphic sites in a genome among a set of breeding lines.

## NEXT-GENERATION SEQUENCE (NGS)

The high demand for low cost sequence data has driven the development of high-throughput sequencing (or next-generation sequencing) technologies that can produce 1000 or millions of sequences concurrently. Next-generation sequencing (NGS) relies on massively parallel sequencing and imaging techniques to yield several 100s of millions to several 100s of billions of DNA bases per run (Shendure and Ji, 2008). Several NGS platforms, such as Roche 454 FLX Titanium (Thudi et al., 2012), Illumina MiSeq and HiSeq2500 (Bentley et al., 2008), Ion Torrent PGM (Rothberg et al., 2011), have been developed and used recently (Deschamps et al., 2012; Quail et al., 2012). High-throughput sequencing technologies are intended to lower the cost of DNA sequencing beyond what is possible with standard dye-terminator methods (Schuster, 2008). In ultra-high-throughput sequencing as many as 500,000 sequencing-by-synthesis operations may be run in parallel (Quail et al., 2012).

All NGS strategies follow a similar protocol for DNA template preparation, where universal adapters are ligated at both ends of randomly sheared DNA fragments. They also rely on the cyclic interrogation of millions of clonally amplified DNA molecules immobilized on a synthetic surface to generate up to several billions of sequences in a massively parallel fashion. Sequencing is performed in an iterative manner, where the incorporation of one or more nucleotides is followed by the emission of a signal and its detection by the sequencer (Metzker, 2010). Most NGS platforms are able to generate reliable sequences and display near perfect coverage behavior on GC-rich, neutral and moderately AT-rich genomes. However, there are key differences between the quality of that data and the applications it will support (Quail et al., 2012).

For Illumina NGS sequencers, DNA molecules and primers are first attached on a slide and amplified with polymerase so that local clonal DNA colonies are formed. To determine the sequence, four types of reversible terminator bases (RT-bases) are added and non-incorporated nucleotides are washed away. A camera takes images of the fluorescently labeled nucleotides, then the dye, along with the terminal 3′ blocker, is chemically removed from the DNA, allowing for the next cycle to begin. Unlike pyrosequencing, the DNA chains are extended one nucleotide at a time and image acquisition can be performed at a delayed moment, allowing for very large arrays of DNA colonies to be captured by sequential images taken from a single camera (Mardis, 2008). NGS can produce ultra-high throughput sequence data on an unparalleled scale compared to Sanger sequencing (Pareek et al., 2011).

NGS technologies commercialized by Illumina generate shorter reads, ranging from 50 to 300 bp, with sequencing throughputs ranging from 1.5 to 600 Gbp depending on the platform being used. Several instruments are commercialized by Illumina, ranging from the bench top MiSeq sequencer to the high-throughput HiSeq2500 sequencer. The Illumina sequencing technology combines clonal amplification of a single DNA molecule with a cyclical sequencing-by-synthesis approach. The PCR amplification is performed using a solid phase amplification protocol to generate up to 1,000 copies of an original molecule of DNA, grouped together into a cluster. Sequencing is performed with proprietary reversible fluorescent terminator deoxyribonucleotides, in a series of cycles consisting of single base extension, fluorescence detection (where the nature of the signal is used to determine the identity of the base being incorporated) and cleavage of both the fluorescent label and of the chemical moieties at the 3′ hydroxyl position to allow for the next cycle to occur (Deschamps et al., 2012).

The application of NGS technologies highlights the striking impact of these massively parallel platforms on genotyping, which have expanded from previously focused readouts from a variety of DNA preparation protocols to a genome-wide scale and have fine-tuned their resolution to single base precision (Kilian and Graner, 2012). NGS has also enabled novel applications, such as the sequencing of ancient DNA samples, and has substantially widened the scope of metagenomic analysis of environmentally derived samples (Mardis, 2008). Based on the accuracy, lower cost, higher throughput and assay simplicity (Gupta et al., 2008), NGS technologies have been recently used for whole genome sequencing and for resequencing projects where the genomes of several specimens are sequenced to discover large numbers of SNPs for exploring the diversity within species, constructing haplotype maps and performing genome-wide association studies (GWAS; Elshire et al., 2011). Multiplex sequencing has also been accomplished by tagging randomly sheared DNA fragments from different samples with unique, short DNA sequences (barcodes) and pooling samples into a single sequencing channel (Craig et al., 2008). This approach (random DNA shearing followed by barcode tagging) has been used to rapidly determine the complete chloroplast genome sequences of spruce and several pine species and for discovery and mapping of genomic SNPs in rice (Cronn et al., 2008; Huang et al., 2009; Elshire et al., 2011).

## GENOTYPING-BY-SEQUENCING (GBS)

**FIGURE 1:**
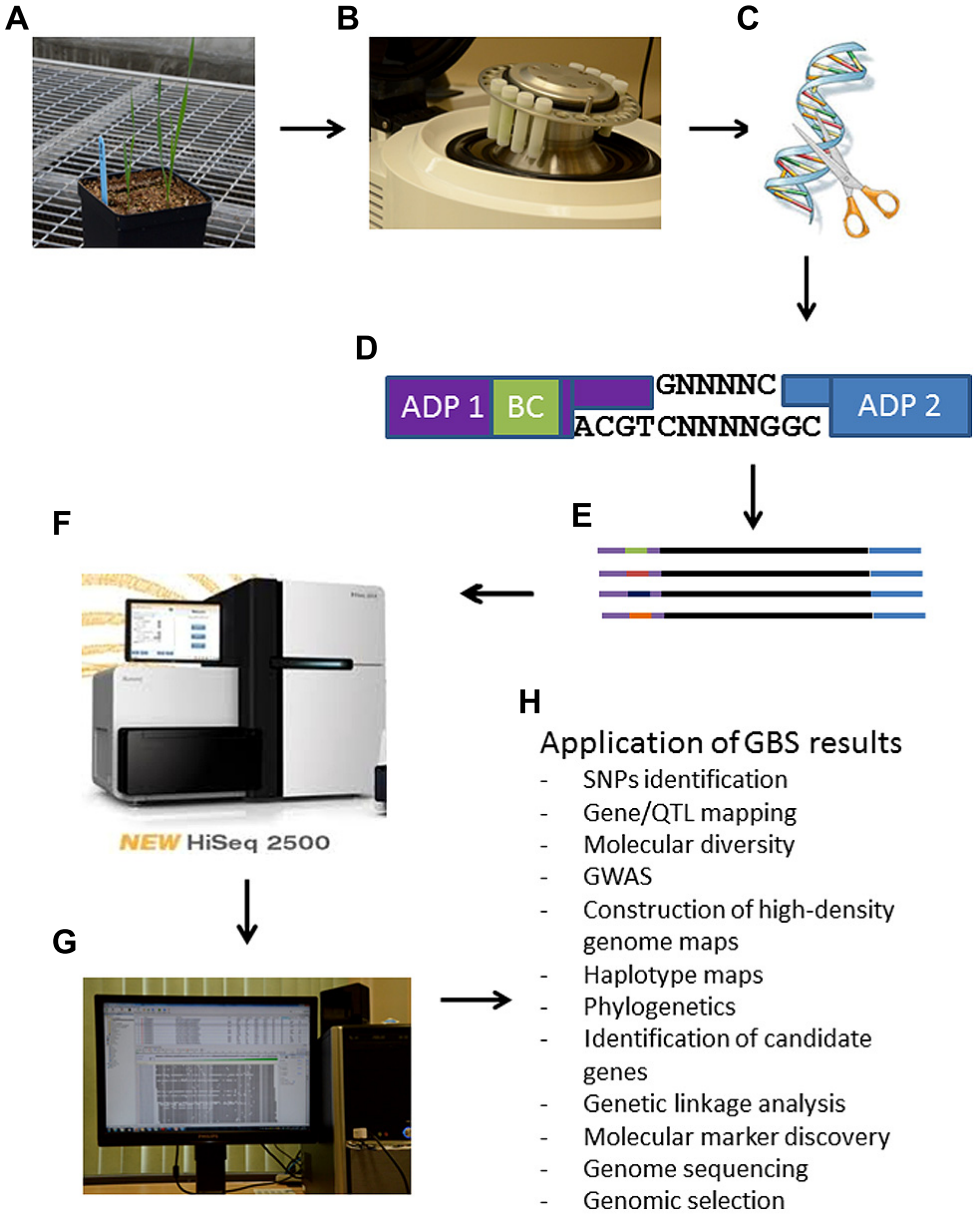
**Schematic steps of the genotyping-by-sequencing (GBS) protocol for plant breeding.** Panel **(A)**: tissue is obtained from any plant species as depicted here a young triticale plant; Panel **(B)**: ground leaf tissues for DNA isolation, quantification and normalization. At this step it is important to prevent any cross-contamination among samples; Panel **(C)**: DNA digestion with restriction enzymes; Panel **(D)**: ligations of adaptors (ADP) including a bar coding (BC) region in adapter 1 in random *PstI-Mse*I restricted DNA fragments; Panel **(E)**: representation of different amplified DNA fragments with different bar codes from different biological samples/lines. These fragments represent the GSB library; Panel **(F)**: analysis of sequences from library on a NGS sequencer; Panel **(G)**: bioinformatic analysis of NGS sequencing data; Panel **(H)**: possible application of GBS results.

Advances in NGS have driven the costs of DNA sequencing down to the point that GBS is now feasible for high diversity and large genome species (Elshire et al., 2011). GBS is a simple highly multiplexed system for constructing reduced representation libraries for the Illumina NGS platform developed in the Buckler lab (Elshire et al., 2011). It generates large numbers of SNPs for use in genetic analyses and genotyping (Beissinger et al., 2013). Key components of this system include low cost, reduced sample handling, fewer PCR and purification steps, no size fractionation, no reference sequence limits, efficient barcoding and easiness to scale up (Davey et al., 2011). GBS is becoming increasingly important as a cost-effective and unique tool for genomics-assisted breeding in a range of plant species. **Figure 1** simplifies the GBS technology by summarizing the steps needed for any plant species and some potential application of the results.

GBS combined with genome-independent imputation provides a simple and efficient method for genetic map construction in any pseudo-testcross progeny (Ward et al., 2013). The GBS method offers a greatly simplified library production procedure more amenable to use on large numbers of individuals/lines (Elshire et al., 2011). A two-enzyme (*PstI/Msp*I) GBS protocol, which provides a greater degree of complexity reduction and uniform library for sequencing than the original protocol using *Ape*KI, has now been developed and applied to both wheat and barley (Poland et al., 2012a). Sonah et al. (2013) described a modified library preparation protocol, in which selective amplification is used to increase both the number of SNPs called and their depth of coverage, resulting in a high efficiency to allow an important reduction in per sample cost.

Two different GBS strategies have been developed with the Ion PGM system (Poland et al., 2012a). (A) Restriction enzyme digestion, in which no specific SNPs have been identified and ideal for discovering new markers for MAS programs. The complexity of the genome under this approach is reduced by digesting the DNA with one or two selected restriction enzymes prior to the ligation of the adapters. (B) Multiplex enrichment PCR, in which a set of SNPs has been defined for a section of the genome. This approach uses PCR primers designed to amplify the areas of interest.

The value of sequencing restriction site associated genomic DNA (RAD) for high density SNP discovery and genotyping was first demonstrated by Baird et al. (2008). Increased efficiency and cost benefits were realized by incorporating a multiplex sequencing strategy that uses an inexpensive barcoding system. Barcodes are included in one of the adapter sequences, and their locations, just upstream of the RE cut-site in genomic DNA, eliminate the need for a second Illumina sequencing (“indexing”) read. The barcoding strategy is similar to RAD but modulation of barcode nucleotide composition and length results in fewer sequence phasing errors (Baird et al., 2008). Compared to the RAD method, GBS is substantially less complicated; generation of restriction fragments with appropriate adapters is more straightforward, single-well digestion of genomic DNA and adapter ligation results in reduced sample handling, there are fewer DNA purification steps, and fragments are not size selected. Costs can be further reduced via shallow genome sampling coupled with imputation of missing internal SNPs in haplotype blocks.

GBS was originally developed for high resolution association studies in maize and, like RAD, has been extended to a range of species with complex genomes. Unlike other high density genotyping technologies which have mainly been applied to general interest “reference” genomes, the low cost of GBS makes it an powerful approach on discovering and genotyping SNPs in a variety of crop species and populations. As a technically simple, highly multiplexed technology, GBS is suitable for population studies, germplasm characterization, plant genetics, and breeding in diverse crops and it has widely been applied in many large crop genomes to saturate the mapping and breeding populations with 10–100s of 1000s of SNP markers (Poland et al., 2012a).

Construction of GBS libraries is based on reducing genome complexity with restriction enzymes (REs; Elshire et al., 2011). This approach is simple, quick, extremely specific, highly reproducible, and may reach important regions of the genome that are inaccessible to sequence capture approaches. By choosing appropriate REs, repetitive regions of genomes can be avoided, and lower copy regions can be targeted with two to three fold higher efficiency (Gore et al., 2007), which tremendously simplifies computationally challenging alignment problems in species with high levels of genetic diversity. The GBS procedure is demonstrated with maize and barley recombinant inbred populations where roughly 200,000 and 25,000 sequence tags were mapped, respectively (Elshire et al., 2011).

## APPLICATION OF GBS IN PLANT BREEDING

Genotyping-by-sequencing is an ideal platform for studies ranging from single gene markers to whole genome profiling (Poland and Rife, 2012). One of the most powerful applications of GBS is in the field of plant breeding. GBS provides a rapid and low-cost tool to genotype breeding populations, allowing plant breeders to implement GWAS, genomic diversity study, genetic linkage analysis, molecular marker discovery, and genomic selection (GS) under a large scale of plant breeding programs. There is no requirement for a priori knowledge of the species genomes as the GBS method has been shown to be robust across a range of species and SNP discovery and genotyping are completed together (Poland and Rife, 2012; Narum et al., 2013).

As GWAS require 100s of 1000s to millions of markers to generate sufficient information and coverage, the emergence of NGS technologies has greatly improved such marker resolution (Edwards and Batley, 2010). Recently, GBS through the NGS approach has been used to resequence collections of recombinant inbred lines (RILs) to analyze and map various traits of interest in specific breeding programs (Deschamps et al., 2012). More and more crops, such as maize, wheat, barley, rice, potato, and cassava, have been optimized by GBS for the efficient, low-cost and large scales of genome sequencing (Poland and Rife, 2012; van Poecke et al., 2013). A collection of 5,000 RILs have been resequenced using a restriction endonuclease-based approach and the Illumina sequencing technology, which generated a total of 1.4 million SNPs and 200,000 indels in maize (Gore et al., 2009). A comprehensive genotyping of 2,815 maize inbred accessions showed that 681,257 SNP markers are distributed across the entire genome, in which some SNPs are linked to the known candidate genes for kernel color, sweetness, and flowering time (Romay et al., 2013). A set of 205,614 SNPs have been identified after resequencing 31 soybean genotypes, providing a valuable genomic resource for soybean breeding programs (Lam et al., 2010). In potato, 12.4 gigabases of high-quality sequence data and 129,156 sequence variants have been identified, which are mapped to 2.1 Mb of the potato reference genome with a median average read depth of 636 per cultivar (Uitdewilligen et al., 2013).

GBS has been shown to be a valid tool for genomic diversity studies (Fu and Peterson, 2011; Lu et al., 2013; Fu et al., 2014). For example, Fu and Peterson (2011) applied the Roche 454 GS FLX Titanium technology with reduced genome representation and advanced bioinformatics tools to analyze the genetic diversity of 16 diverse barley landraces, discovered 2,578 contigs, and 3,980 SNPs, and confirmed a key geographical division in the cultivated barley gene pool. Lu et al. (2013) developed a network-based SNP discovery protocol to enhance the diversity analysis of 540 switchgrass plants sampled from 66 populations and revealed informative patterns of genetic relationship with respect to ecotype, ploidy level, and geographic distribution. The GBS protocol was used to analyze genetic diversity of 24 diverse yellow mustard accessions, in which roughly 1.2 million sequence reads (total about 392 million nucleotides) were generated, 512 contigs, and 828 SNPs were identified (Fu et al., 2014). Diversity analysis of these yellow mustard SNPs revealed that 26.1% of total variation resided among landrace, cultivar, and breeding lines and 24.7% between yellow-seeded and black-seeded germplasm.

Identification of high density SNP markers through GBS to construct genetic linage maps has a great value for numerous applications in plant breeding. In *Arabidopsis*, Schneeberger et al. (2009) sequenced, via whole genome shotgun sequencing on the Illumina platform, a pool of 500 F2 plants generated by crossing a recessive ethane methyl sulfonate (EMS)-induced Col-0 mutant characterized by slow growth and light green leaves, with a wild type L*er* (Landsberg *erecta*) line. Spindel et al. (2013) used a 384 plex GBS protocol to add 30,984 SNP markers to an *indica* ×*japonica* mapping population consisting of 176 rice recombinant inbred lines and mapped the recombined hot and cold spots and quantitative trait loci (QTLs) for leaf width and aluminum tolerance. After the efficiency of multiplexed SNP genotyping for diversity, mapping and breeding applications were evaluated, Thomson et al. (2012) demonstrated that 384 plex SNP genotyping on the BeadXpress platform is a robust and efficient method for marker genotyping and mapping in rice (Heffner et al., 2009; Huang et al., 2009; Jannink et al., 2010). GBS was applied to bread wheat, resulting in the incorporation of 1000s of markers in the bread wheat map (Poland et al., 2012a). The high resolution of SNP markers were identified in barley and the GBS mapping data were used to confirm that the semi-dwarfing gene (*ari-e*) is located on barley chromosome 5H (Liu et al., 2014). Construction of a GBS linkage map using the sequence-based markers leads to the RAD technique (Baird et al., 2008), which has been used in barley QTL analysis (Chutimanitsakun et al., 2011).

By integrating molecular markers and genotyping of large populations, GBS is an excellent platform for plant breeding applications even in the absence of reference genome sequences or without previous DNA polymorphism discovery. The GBS approach has been shown to be suited to genetic analysis and marker development of rapeseed, lupin, lettuce, switchgrass, soybean, and maize (Bus et al., 2012; Truong et al., 2012; Yang et al., 2012; Lu et al., 2013; Sonah et al., 2013). With Illumina genome analyzer, Varala et al. (2011) identified 4294 to 14550 SNPs between four soybean accessions and the reference and indicated that the *Mse*I digestion of soybean genomic DNA followed by high throughput sequencing provides a rapid and reproducible method for generating SNP markers. High-throughput SNP discovery and genotyping in durum wheat have been investigated from 92 RILs derived from a cross between the two elite cultivars (Mantovani et al., 2008). The application of GBS on a large collection of autotetraploid potato cultivars were studied with Illumina HiSeq2000 and the alleles strongly associating with maturity and flesh color were identified (Uitdewilligen et al., 2013).

Compared to traditional MAS, GS is a novel approach which combines molecular markers with phenotypic and pedigree data to increase the breeding accuracy on genotypic values (Heffner et al., 2009). Theoretical and applied studies on GS show great promise to accelerate the rate of developing new crop varieties. GS through the GBS approach stands to be a major supplement to traditional crop improvement and it is a very important feature to move the genomics-assisted breeding into commercial crops with large and complex genomes (Poland and Rife, 2012). One premise of GBS applications is the development of genome-wide molecular markers with high density and low cost (Heffner et al., 2009, 2010; Jannink et al., 2010). GBS approach on barley and wheat study (Poland et al., 2012a) provides a powerful method of developing high density markers in species without a reference genome while providing valuable tools for anchoring and ordering physical maps and whole genome shotgun sequence. Poland et al. (2012b) used GBS to discover 41,371 SNPs in a set of 254 advanced breeding lines from CIMMYT’s semiarid wheat breeding program. Ward et al. (2013) reported that the high marker density allows the identification of genomic regions with segregation distortion in *Rubus idaeus*, which may help to identify deleterious alleles that are the basis of inbreeding depression in that species. An efficient GBS approach has been developed to catalog SNPs both within the mapping population and among diverse African cassava varieties, allowing the improvements of MAS programs on disease resistance and nutrition in cassava (Prochnik et al., 2012).

Although GBS offers a novel approach on enhancing the efficiency and capacity of plant breeding, some potential drawbacks have been identified under its applications, which seems not unique to this technique. A major challenge encountered by all genotyping methods has been the difficulty to align true alleles of each single locus in large, complex, polyploidy genomes. Among all the tools available, however, GBS is the one offering the higher potential to resolve the issue. As exemplified by Huang et al. (2014), alleles in hexaploid oat can be distinguished after extensive analyses of sequence data through two different bioinformatics pipelines, suggesting the data analysis algorithms may now represent the limiting factor to ascertain alleles at each single locus in a large polyploidy genome rather than GBS itself given sufficient depth of sequence is available.

The reduction in genome complexity using restriction enzymes in the GBS protocol means that, in case of any mutation at the restriction site, the genomic DNA of this region is not available to be PCR amplified and consequently the SNPs of this region will become unavailable. In the worst case of this scenario, a heterozygote gene may appear as homozygous. However, this is a drawback shared by all the different methods involving reduction in genome complexity based on the utilization of restriction sites. No scientist is ready to sacrifice the high throughput of these methods to move back to RFLP-based protocols. The feasibility of reduced representation and highly multiplexed GBS strategy was demonstrated in the complex genomes of maize and barley via a simple procedure targeting regions flanking restriction endonuclease sites (Elshire et al., 2011).

Epigenetic studies have revealed the importance of differential DNA methylation in numerous biological systems. Two restriction enzymes [a rare cutter, *Pst*I (CTGCAG), and a frequent cutter, *Msp*I (CCGG)] were employed to improve the reduction of genome complexity in barley and wheat (Poland et al., 2012a). It may have been overlooked that the activity of *Msp*I is inhibited when the DNA is methylated at the external “C.” In epigenetic studies, however, the activity of the isoschizomer *Hpa*II is inhibited by methylation at any of two “C.” Because of the wide utilization of *Hpa*II, the methylation of the internal “C” in epigenetic regulation studies seems to be much more important than the methylation of the external “C.” Therefore, the possibility that developmental responses in plants may affect the SNP identification when using the enzyme *Msp*I cannot be ignored, but is likely reduced.

Orphan plant species without a known genomic sequence represent the vast majority of crops over the world. The GBS protocol for wheat and barley and subsequent genetic analyses (Poland et al., 2012a) were carried out when a draft genomic sequence was not available yet. An available reference genome can simplify the data analyses, but it is not essential in GBS, indicating a great advantage of the GBS technique in accelerating plant breeding and crop improvement. This reality has been confirmed with the recent GBS applications on different oat accessions (Huang et al., 2014). The depth of genomic sequencing is important to identify stable and representative SNPs which can be generated to improve crop genotypes. Huang et al. (2014) also demonstrate the importance of the bioinformatic pipeline to fully exploit the GBS datasets, which is likely more critical in orphan plant species.

## PERSPECTIVES

Genotyping-by-sequencing is a novel application of NGS protocols for discovering and genotyping SNPs for crop improvement. The low cost of GBS makes it an attractive approach to saturate the mapping and breeding populations with a high density of SNP markers. Successive improvements of the sequencing chemistries and base-calling software will allow NGS technologies to deliver higher sequencing throughputs per run, which in turn enables deeper multiplexing for a fixed average sequencing depth per sample. As the amount and quality of sequence information generated per run keeps increasing, which allows even higher plexing and lower costs per samples, GBS has become a cost-competitive alternative to other whole genome genotyping platforms. It can be anticipated that high density of SNP markers from NGS will be extensively applied to GWAS, MAS, and GS. Plant breeders will be able to sequence even large crop genomes and establish high density of genetic linkage maps from breeding populations. Future applications of GBS to crop improvement may allow plant breeders to conduct MAS or GS on a novel germplasm or species without first having to develop any prior molecular tools. As the sequence-based genotyping is available for whole range of genomic studies, GBS will stand to be one of major components in plant genetics and breeding.

## Conflict of Interest Statement

The authors declare that the research was conducted in the absence of any commercial or financial relationships that could be construed as a potential conflict of interest.
